# Multimodal fusion model for diagnosing mild cognitive impairment in unilateral middle cerebral artery steno-occlusive disease

**DOI:** 10.3389/fnagi.2025.1527323

**Published:** 2025-02-12

**Authors:** Ziyi Yuan, Zhaodi Huang, Chaojun Li, Shengrong Li, Qingguo Ren, Xiaona Xia, Qingjun Jiang, Daoqiang Zhang, Qi Zhu, Xiangshui Meng

**Affiliations:** ^1^School of Medicine, Cheeloo College of Medicine, Shandong University, Jinan, China; ^2^Department of Radiology, Meng Chao Hepatobiliary Hospital of Fujian Medical University, Fuzhou, Fujian, China; ^3^College of Artificial Intelligence, Nanjing University of Aeronautics and Astronautics, Key Laboratory of Brain-Machine Intelligence Technology, Ministry of Education, Nanjing, China; ^4^Department of Radiology, Qilu Hospital (Qingdao), Cheeloo College of Medicine, Shandong University, Qingdao, China; ^5^Medical Imaging and Engineering Intersection Key Laboratory of Qingdao, Qingdao, China

**Keywords:** middle cerebral artery, stenosis, multimodality imaging, mild cognitive impairment, Montreal cognitive assessment

## Abstract

**Objectives:**

To propose a multimodal functional brain network (FBN) and structural brain network (SBN) topological feature fusion technique based on resting-state functional magnetic resonance imaging (rs-fMRI), diffusion tensor imaging (DTI), 3D-T1-weighted imaging (3D-T1WI), and demographic characteristics to diagnose mild cognitive impairment (MCI) in patients with unilateral middle cerebral artery (MCA) steno-occlusive disease.

**Methods:**

The performances of different algorithms on the MCI dataset were evaluated using 5-fold cross-validation. The diagnostic results of the multimodal performance were evaluated using t-distributed stochastic neighbor embedding (t-SNE) analysis. The four-modal analysis method proposed in this study was applied to identify brain regions and connections associated with MCI, thus confirming its validity.

**Results:**

Based on the fusion of the topological features of the multimodal FBN and SBN, the accuracy for the diagnosis of MCI in patients with unilateral MCA steno-occlusive disease reached 90.00%. The accuracy, recall, sensitivity, and F1-score were higher than those of the other methods, as was the diagnostic efficacy (AUC = 0.9149).

**Conclusion:**

The multimodal FBN and SBN topological feature fusion technique, which incorporates rs-fMRI, DTI, 3D-T1WI, and demographic characteristics, obtains the most discriminative features of MCI in patients with unilateral MCA steno-occlusive disease and can effectively identify disease-related brain areas and connections. Efficient automated diagnosis facilitates the early and accurate detection of MCI and timely intervention and treatment to delay or prevent disease progression.

## 1 Introduction

Intracranial arterial stenosis (ICAS) is an independent risk factor for cerebral ischaemia. Stenosis of the middle cerebral artery (MCA) is the most frequent subtype of ICAS (Zhang L. et al., 2022). Persistent arterial stenosis can lead to intracranial ischaemic damage, resulting in brain atrophy and secondary neurodegeneration, which may affect cognitive function. Mild cognitive impairment (MCI) is an intermediate state between normal aging and dementia (Petersen et al., 2018; Cheng et al., 2017; Langa and Levine, 2014). Various scales are used to diagnose MCI, such as the Mini-Mental State Examination (MMSE), Montreal cognitive assessment (MoCA), and AD8 (Pinto et al., 2019; Nasreddine et al., 2005; Usarel et al., 2019). Different scales use slightly different criteria and methods to assess cognitive function, which may result in different diagnostic results (Zhuang et al., 2021). In addition, the scale assessment process is limited by a certain degree of subjectivity because the scoring criteria and results may be affected by the assessor’s personal experience and bias, leading to incorrect or missed diagnoses. The characteristics and symptoms of MCI vary among different populations. For example, factors such as age, cultural background, and education level may affect the accuracy of assessment results. Therefore, it is of great clinical significance to investigate intelligent diagnostic and analytical methods for MCI as crucial interventions to ensure early diagnosis and timely treatment.

With the development of imaging technology, multimodal magnetic resonance imaging (MRI) has provided objective supplementary disease biomarkers for the computer-aided diagnosis of MCI. Structural MRI shows specific cerebral gray and white matter atrophy (Pennanen et al., 2004). Resting-state functional MRI (rs-fMRI) indirectly detects neural activity in the brain based on blood oxygen level-dependent (BOLD) signals and can detect abnormalities in brain function in patients with MCI (Li et al., 2016; Liu et al., 2016). Diffusion tensor imaging (DTI) is widely used to study the orientation and integrity of white matter fiber tracts by measuring the Brownian motion of water molecules in neural tissues (Le Bihan, 2003), indirectly reflecting tissue microstructure and pathological changes (Jiang et al., 2006). Significant differences in anisotropy scores (FA) and mean diffusivity (MD) have been found in the white matter of patients with MCI compared to normal subjects (Sexton et al., 2011; Yu et al., 2017; Shim et al., 2017).

Despite the utility of the techniques, learning disease features and identifying imaging markers by using a single modality have limitations. Multimodal MRI can integrate complementary information from different modalities, thereby improving disease diagnosis by detecting subtle structural alterations in the brain more accurately than with a single modality. Therefore, several studies investigating MCI have used combinations of functional and structural connectivity networks, with results indicating that network features based on multimodal images are advantageous for the diagnosis of MCI (Zhu et al., 2014; Zhang et al., 2011; Song et al., 2023). For example, studies have shown that the integration of multiple modalities, such as genetic, epigenomic, and neuroimaging data, using hyper-graph-based sparse canonical correlation analysis (HGSCCA) can extract meaningful biomarkers related to MCI (Shao et al., 2021). Joint neuroimaging synthesis representation learning (JSRL) has been proposed for conversion using incomplete multi-modal neuroimaging data and has shown superior performance for MCI cross-database synthesis compared to several state-of-the-art methods (Liu et al., 2022). In addition, feature selection methods, such as a multi-classification prediction model based on fusing multi-modal features, have been developed to accurately diagnose and predict the progression of MCI. The proposed feature selection method with a multikernel support vector machine (MK-SVM) showed better classification performance than state-of-the-art multimodality-based methods (Hao et al., 2020). In another study, Lei et al. (2021) constructed a functional brain network (FBN) and structural brain network (SBN) based on rs-fMRI and DTI, respectively, and used an automatic weighted centralized multitasking learning framework to integrate these structural and functional connectivity features, achieving diagnostic accuracies higher than 84.80% between normal controls and patients with subjective cognitive impairment with MCI. This suggests that connected networks based on multimodal images have significant potential for MCI classification and diagnosis.

Studies have demonstrated that extracranial arterial stenosis is an independent risk factor for cognitive dysfunction (Huang et al., 2018; Dempsey et al., 2018; Wang et al., 2017). However, few studies have investigated the correlation between intracranial arterial stenosis, particularly MCA stenosis, and cognitive impairment. In additionally, uniform diagnostic imaging criteria for cognitive impairment caused by intracranial vascular stenosis are lacking. Therefore, this study tested a novel fusion technique based on the topological features of multimodal FBN and SBN to improve early diagnosis of MCI in patients with unilateral MCA steno-occlusive disease. The main contributions of the proposed method are summarized as follows:

• We developed a multimodal framework that combines rs-fMRI, DTI, three-dimensional-T1-weighted imaging (3D-T1WI), and demographic data, leveraging the complementary strengths of these modalities for diagnosing MCI in patients with unilateral MCA steno-occlusive disease.
• A topological feature fusion technique was introduced that preserves SBN and FBN properties while using attention mechanisms to integrate multi-channel topological features and identify MCI-related brain regions and connections.
• Our method achieved superior performance, with an accuracy (ACC) of 90.00% and an area under the receiver operating characteristic (ROC) curve (AUC) of 0.9149, surpassing existing techniques and providing an effective tool for early MCI detection and intervention.

## 2 Materials and methods

### 2.1 Participants

Forty patients with unilateral MCA steno-occlusive disease, diagnosed based on magnetic resonance angiography (MRA) between January 2017 and August 2023, were recruited and divided into two groups based on MoCA results. The MCI and non-MCI (NMCI) groups comprised 11 and 29 patients, respectively. According to Chinese MoCA norms (Lu et al., 2011), the level of cognitive impairment is defined by the following scores: ≤ 13 for illiterate individuals, ≤ 19 for individuals with 1–6 years of education, and ≤ 24 for those with 7 or more years of education. The inclusion criteria were as follows: (a) asymptomatic or subjective memory decline; (b) unilateral MCA stenosis > 70%; (c) absence of stroke, TIA, or dementia; (d) right-handed; (e) ability to complete the MRI examination with a qualifying high-resolution MRI image; (f) no history of drug use that could affect cognitive function; and (g) normal-appearing white matter (normal brain parenchymal signals or lacunar infarcts < 3 mm in diameter on T2-weighted and fluid attenuated inversion recovery [FLAIR] sequences). The exclusion criteria were as follows: (a) other cerebral artery stenosis ≥ 30%; (b) severe visual or auditory impairment preventing completion of cognitive function assessment; (c) history of severe systemic or neuropsychiatric diseases; (d) history of frequent dizziness and headache; (e) history of acute or chronic cerebral infarction, bleeding, tumor, infectious disease, or metabolic disease detected by MRI; (f) history of drug or alcohol dependence during the last 6 months; and (g) contraindications for MRI. This study was approved by the Medical Ethics Committee of Q Hospital, and informed consent was obtained from all participants. The detailed demographic characteristics of the participants are presented in Table 1.

**TABLE 1:** Demographic characteristics of the subjects.

	NMCI (*n* = 29)	MCI (*n* = 11)	χ 2/T/Z	*p*-value
Age (years, median, IQR)	59.3 (16.00)	64.2 (2.00)	−1.352	0.176^c^
Sex (female, %)	16 (55.17%)	6 (54.55%)	0.000	1.000^a^
Education (years, mean ± SD)	9.03 ± 4.64	9.18 ± 2.04	−0.139	0.890^b^
Hypertension (%)	24 (82.76%)	8 (72.73%)	0.071	0.791^a^
Diabetes (%)	13 (44.83%)	3 (27.27%)	0.423	0.515^a^
Hyperlipidemia (%)	13 (44.83%)	7 (63.64%)	1.129	0.288^a^
Current smoker (%)	6 (20.69%)	2 (18.18%)	0.000	1.000^a^
Current drinker (%)	6 (20.69%)	3 (27.27%)	0.000	0.983^a^
Severe stenosis or occlusion side (right, %)	13 (44.83%)	7 (63.64%)	1.129	0.288^a^

*^a^*Fisher’s exact test.

*^b^*Independent-samples *t*-test.

*^c^*Mann–Whitney U test. *p*-value significant cut-off 0.05. IQR, interquartile range; SD, standard deviation.

Hypertension was defined as a self-reported diagnosis by a physician, antihypertensive medication use, or systolic or diastolic blood pressure ≥ 140 or ≥ 90 mmHg, respectively. Diabetes was defined as a self-reported history of antidiabetic medication use or glycated hemoglobin A1C level ≥ 6.5%. Hyperlipidaemia was defined as a history of hyperlipidemia, a clinical diagnosis of hyperlipidaemia during hospitalization, or the use of lipid-lowering medication (American Diabetes Association, 2020; Israelsson et al., 2017; Jaraj et al., 2016). A current smoker was defined as someone who smoked >100 cigarettes in their lifetime and was currently smoking cigarettes at the time of the survey (Adeloye et al., 2019). A current drinker was defined as someone who consumed at least one alcoholic beverage per week during the past month.

### 2.2 MRI data acquisition

Brain MRI was performed using a 3.0T MRI scanner (Ingenia; Philips Medical Systems, Netherlands). A matched head coil with foam padding and earplugs were used to reduce head motion and scanner noise. The scanning sessions were performed using the following parameters: (1) T2-weighted imaging (T2WI): 18 axial slices, 6-mm slice thickness with a 1-mm gap, repetition time/time to echo (TR/TE) = 2,369/107 ms, matrix = 352 × 352; (2) T2WI- FLAIR: 18 axial slices, 6-mm slice thickness with a 1-mm gap, TR/TE = 7,000/125 ms, matrix = 288 × 163; (3) diffusion weighted imaging (DWI): 18 axial slices, 6-mm slice thickness with a 1-mm gap, TR/TE = 2,235/76 ms, matrix = 176 × 134; (4) 3D-T1WI: 170 sagittal slices, 1-mm slice thickness with no gap, TR/TE = 6.7/3.0 ms; (5) DTI: (70 axial slices, 2 mm slice thickness with no gap, TR/TE = 4,900/95 ms, matrix = 122 × 110, b values = 1,000 s/mm^2^) in 32 directions; and (6) rs-fMRI: 32 axial slices, 4-mm slice thickness with a 0.5-mm gap, 240 time points, TR/TE = 2,000/30 ms, flip angle = 90°, field of view = 230 mm^2^ × 230 mm^2^, data matrix = 68 × 66, voxel = 3.5 mm^3^ × 3.5 mm^3^ × 4 mm^3^.

### 2.3 Data preprocessing

(1) rs-fMRI processing: All rs-fMRI data were pre-processed using the DPARSF toolbox (Chao-Gan and Yu-Feng, 2010). Following the general fMRI preprocessing pipeline, the serialized data were split into several pieces and adjusted to the echo-planar imaging template to correct and rectify the initial image (Zhang J. et al., 2022; Zhu et al., 2024). Detrending was used to reduce the effects of head motion and interference from the cerebrospinal fluid (CSF) and white matter. After linear detrending, the data were filtered using a typical time bandpass filter to reduce low-frequency drift and high-frequency physiological noise. Next, the motion parameters, global mean signal, white matter, and CSF were employed as interference covariates to reduce the effects of head movement and non-neuronal blood oxygenation level dependent fluctuations. After processing, we employed an automated anatomical labeling (AAL) atlas (Craddock et al., 2012) to partition the rs-fMRI date into 90 brain regions, each containing blood oxygen level signals at 240 time points, with feature dimensions of 90 × 240.

(2) DTI processing: DTI distortions were first corrected using the FSL-based PANDA toolbox (Cui et al., 2013). The processing procedure inclouded the following steps: b0-based brain extraction utilizing the bet function and correction for eddy currents and head motion employing the eddy_correct function with b0 serving as the reference volume. Based on each subject’s co-registered T1 images, TrackVis was used to obtain fiber images using a deterministic tracking method, and the anatomic areas were defined using AAL conventions. Finally, the number of fibers was used to measure the structural connectivity with feature dimensions of 90 × 90.

(3) 3D-T1WI processing: The CAT12 toolbox of Statistical Parametric Mapping (SPM12) (Ashburner and Friston, 2005) was used to segment the 3D-T1WI. Regions of interest (ROIs) refer to specific areas of the brain selected for detailed analysis, which are often based on prior knowledge or anatomical templates. In this case, the AAL template was used to extract the volume of the 116 ROIs from the segmented gray matter. Given that we focused exclusively on the brain, brain regions 91–116 were removed as features, resulting in feature dimensions of 90 × 1. The last 26 brain regions were removed to exclude those that were less relevant to the specific analysis, thus ensuring a more focused and meaningful feature set for the given task.

(4) Demographic characteristic processing: Participant information was coded with dimensions of 90 × 9.

### 2.4 Multimodal imaging technique based on rs-fMRI, DTI, 3D-T1WI, and demographic characteristics

**FIGURE 1:**
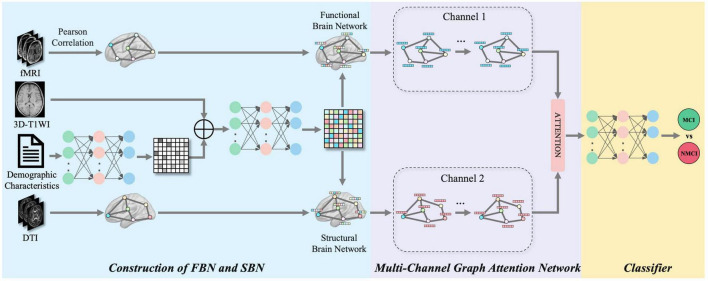
The framework of the proposed multimodal brain networks fusion for brain disease diagnosis. Our framework can be divided into three parts: construction of FBN and SBN, multi-channel graph attention network and classifier.

In this study, we proposed a framework that fuses multimodal (four-modality) FBN and SBN topological features, focusing on multimodal classification using four modalities: rs-fMRI, DTI, 3D-T1WI, and demographic characteristics. Figure 1 shows a schematic of the proposed multimodal data fusion and classification system. The construction of FBNs and SBNs plays a key role in generating brain network data with topological properties. A multichannel graph attention network was utilized to extract spatial features from multichannel graph-structured data. The attention mechanism effectively fuses features from different channels, and a multilayer perceptron (MLP) (Rosenblatt, 1958; Zhou et al., 2023) classifier was then applied to classify the extracted features.

**FIGURE 2:**
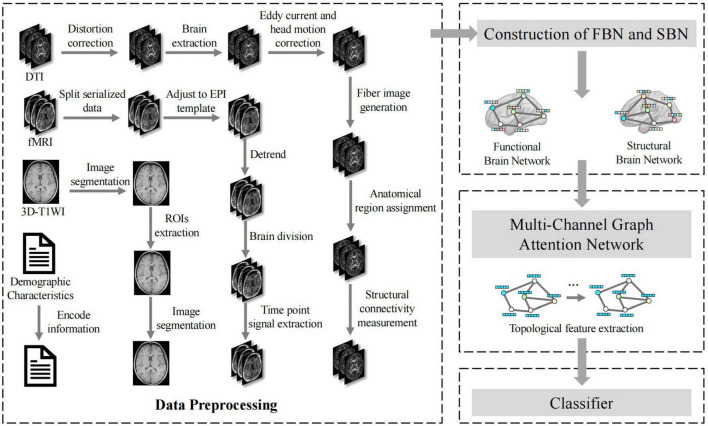
Application flow chart of multimodal brain network construction and feature fusion in MCI diagnosis.

As illustrated in Figure 2, our framework initiated a comprehensive data processing phase that standardizes the fMRI, DTI, and 3D-T1WI data. These processes include distortion correction, brain extraction, eddy current and head motion correction, image segmentation, ROI extraction, and time-point signal extraction, along with the integration of encoded demographic characteristics. Subsequently, FBNs and SBNs were constructed to reveal dynamic functional connectivity and physical connections between brain regions, respectively. A multichannel graph attention network was then employed to extract the topological features from these networks. The network utilizes an attention mechanism to enhance the representation of brain regions associated with MCI and integrates multimodal information to improve the comprehensiveness and accuracy of features. The extracted features were then fed into an optimized classifier, which was designed to enhance the accuracy, recall, specificity, and F1 score for MCI diagnosis, and the model’s diagnostic performance was further assessed through the ROC curve. Figure 2 provides a clear visual representation of the entire process, from data preprocessing to brain network construction to feature extraction and classification, offering a transparent view of how the various components of the multimodal brain network analysis workflow interact and collaborate to effectively diagnose MCI. This integrated approach allows a more comprehensive capture of brain network changes related to MCI, thereby providing a scientific basis for early diagnosis and intervention.

#### 2.4.1 Construction of functional brain networks and structural brain networks

In the diagnosis of diseases, single-modal brain imaging data such as 3D-T1WI, rs-fMRI, and DTI contain complex and unique discriminative information. A vector representation constructed as an Euclidean space is not conducive to data fusion or information extraction. 3D-T1WI can generate static images to obtain information regarding the patient’s body, rs-fMRI reflects changes in brain activity in the temporal dimension, and DTI reflects the physical connectivity of brain intervals in the spatial dimension. In addition, demographic characteristics, including years of education, type of MCA stenosis, sex, age, disease history, and other information comprehensively reflect the background of the subjects. Changes in the brain connectivity patterns are important features of brain disorders. Constructing the brain networks of subjects is a common method for intelligent diagnosis of brain diseases. For each subject, we defined the demographic characteristics matrix *E* = (*e*, *e*,⋯,*e*_*P*_)^*T*^ ∈ ℝ^*P*^, where *P* represents the amount of background information in demographic characteristics. The feature matrix of 3D-T1WI was defined as *T* = (*t*_1_, *t*,⋯,*t*_*N*_)^*T*^ ∈ ℝ^*N*^, where *N* denotes the number of ROIs. We further introduced a multilayer perceptron to transform the dimensions of the demographic characteristic matrix. The process is as follows:


(1) $$\begin{array}{c} E\prime =f_{1}\,\left(w_{1},E\right)+b_{1} \end{array}$$

where *f*_*1*_ is parameterised by the network weights *w*_1_ ∈ ℝ^*N* × *P*^ and the learnable bias term *b*_1_ ∈ ℝ^*N*^. We further performed a preliminary fusion of *E*′ and *T* to obtain the feature F: *F* = *E*′ + *T*. Next, we transformed the dimensions of the fused feature matrix as follows:


(2) $$\begin{array}{c} R=f_{2}\,\left(w_{2},F\right)+b_{2} \end{array}$$

where *f*_*2*_ is parameterised by the network weights *w*_2_ ∈ ℝ^*N*×*N*^ and *b*_2_ ∈ ℝ^*N*^ is the learnable bias term, *R* ∈ ℝ^*N*^. At the same time, we defined the rs-fMRI time-series data for each subject as *X* = (*x*_1_, *x*_2_,⋯,*x*_*N*_)^*T*^ ∈ ℝ^*N*×*M*^, where *N* denotes the number of brain regions and *M* represents the number of consecutive time series points collected. Pearson’s correlation coefficients were calculated for the paired ROIs to measure functional connectivity.


(3) $$\begin{array}{c} c_{i\,j}=\frac{C\,o\,v\,\left(x_{i},x_{j}\right)}{\sigma_{x_{i}}\,\sigma_{x_{j}}} \end{array}$$

where $C\,o\,v\,\left({\vec{x}}_{i},{\vec{x}}_{j}\right)$ denotes the covariance of ${\vec{x}}_{i}$ and ${\vec{x}}_{j}$ and σ denotes the standard deviation. Thus, we obtained the functional connectivity matrix *C* = (*c*_1_, *c*_2_,⋯,*c*_*N*_)^*T*^ ∈ ℝ^*N* × *N*^, where vector ${\vec{c}}_{i}$ denotes functional connectivity feature of the *i^th^* brain region. The DTI feature matrix is *D* = (*d*_1_, *d*_2_,⋯,*d*_*N*_)^*T*^, *d*_*i*_ ∈ ℝ^*N*^, and the values in the matrix reflect the strength of brain interval connectivity. To achieve further fusion of different modalities, we defined the FBN as *G*_*F*_ = (*R*, *C*), and the SBN as *G*_*S*_ = (*R*, *D*).

#### 2.4.2 Multichannel graph attention network

To maintain the topological information of FBNs and SBNs, we developed a method to extract the topological features of brain networks using multi-channel graph attention networks. A multi-channel graph attention network is primarily composed of two graph-attention networks, as shown in Figure 1. The input to each channel is a series of feature vectors of the brain regions and connections. Taking the FBN*G*_*F*_ = (*R*, *C*) as an example, its node feature can be expressed as R = (*r*_1_, *r*_2_,⋯, *r*_*N*_)^*T*^,*r*_*i*_ ∈ ℝ^*N*^, where the number of brain regions and feature dimension are *N*. If brain regions *i* to brain regions *j* have an edge, the brain area concentration coefficient $e_{i\,j}=L\,e\,a\,k\,y\,R\,e\,L\,U\,\left({\vec{a}}^{T}\,\left[W\,r_{i}∥W\,r_{j}\right]\right)$, where a ∈ ℝ^2*N*^ denotes a learnable attention vector, *W* denotes a learnable weight matrix, and ∥ represents the concatenation operation. *LeakyReLU* is the LeakyReLU activation function, where the parameter α is typically set to 0.2, allowing a small gradient for negative input values to prevent neurones from becoming inactive.

We employed a masking mechanism to embed the graph structure from the adjacency matrix *C* into the attention coefficients. Subsequently, attention coefficient *e*_*ij*_ is updated as follows:


(4) $$\begin{array}{c} e_{i\,j}=\left\{ \begin{array}{c} 0,c_{i\,j}=0\\ e_{i\,j},c_{i\,j}>0 \end{array}\right. \end{array}$$

We further applied the SoftMax function to normalize *e*_*ij*_ for neighboring brain regions *j* ∈ *N*_*i*_ of the *i^th^* brain region. The SoftMax function converts the input values into a probability distribution, ensuring that the normalized values sum to 1 across the neighboring regions. The normalized attention coefficient can then be obtained as follows:


(5) $$\begin{array}{c} \alpha_{i\,j}=s\,o\,f\,t\,m\,a\,x\,\left(\frac{e\,x\,p\,\left(L\,e\,a\,k\,y\,R\,e\,L\,U\,\left({\vec{a}}^{T}\,\left[W\,r_{i}∥W\,r_{j}\right]\right)\right)}{\sum_{k\in N_{i}}e\,x\,p\,\left(L\,e\,a\,k\,y\,R\,e\,L\,U\,\left({\vec{a}}^{T}\,\left[W\,r_{i}∥W\,r_{k}\right]\right)\right)}\right) \end{array}$$

The normalized attention coefficient is used to update the brain network features, and the updated features of the *i^th^* brain region are expressed as follows.


(6) $$\begin{array}{c} z_{F}^{i}=\sigma \,\left(\sum_{j\in {𝒩}_{i}}\alpha_{i\,j}\,W\,r_{j}\right) \end{array}$$

#### 2.4.3 Attention mechanism

The attention mechanism plays a pivotal role in feature fusion, because it enables the model to selectively prioritize the most informative features from each embedding. By assigning adaptive weights to different features, the attention mechanism enhances the capacity of the model to discern and leverage complex interactions between brain regions, leading to more refined and accurate classifications. Furthermore, this approach increases the interpretability of the model, as it provides insight into which specific features or regions contribute most significantly to the decision-making process. Using the multi-channel graph attention network, we obtained two feature embeddings: *Z_F_* and *Z_S_*. Considering that the labels of the brain network are related to their pair combinations, we used the attention mechanism (γ_F_, γ_S_) = *att*(Z_F_, Z_S_) to fuse them, where γ_*F*_, γ_*S*_ ∈ ℝ^*N*×1^ represent the attention values of the *i^th^* brain embedded regions *Z_F_* and *Z_S_*, respectively. For the brain region *i*, its embedding in *Z* was *z*. We first transformed the embedding by nonlinear transformation, and subsequently used a shared attention algorithm *q* ∈ ℝ^*N*×1^ to obtain the attention value $\delta_{F}^{i}$ as follows:


$$\delta_{F}^{i}=q^{T}\,t\,a\,n\,h\,\left(W\,{\left(z_{F}^{i}\right)}^{T}+b\right)$$

where *W* is the weight matrix, and *b* is the bias vector. We normalized the attention values using the SoftMax function:


(7) $$\begin{array}{c} w_{F}^{i}=s\,o\,f\,t\,m\,a\,x\,\left(\delta_{F}^{i}\right)=\frac{e\,x\,p\,\left(\delta_{F}^{i}\right)}{e\,x\,p\,\left(\delta_{F}^{i}\right)+e\,x\,p\,\left(\delta_{S}^{i}\right)} \end{array}$$

Similarly, $w_{s}^{i}=s\,o\,f\,t\,m\,a\,x\,\left(\delta_{S}^{i}\right)$, a larger attention weight indicated that the corresponding embedding is more important. For *N* brain regions, there were learnable weights *w*_F_, *w*_S_ ∈ ℝ^*N*×1^, and γ_F_ = *diag*(*w*_F_), γ_S_ = *diag*(*w*_S_). We then combined the embedding output from the multi-channel graph attention network to obtain the final embedding:


(8) $$\begin{array}{c} Z=\gamma_{F}\cdot Z_{F}+\gamma_{S}\cdot Z_{S} \end{array}$$

#### 2.4.4 MLP classifier

MLP, also known as an artificial neural network (ANN), contains an input layer, output layer, and several hidden layers. The hidden layer is fully connected to the input layer. Assuming that the vector of the input layer is x and h(x) is selected as a linear function, the hidden layer is: *g* = *Hx* + ⋅*k*, and the vector y of the output layeris:


(9) $$\begin{array}{c} y=f\,\left(g\,\left(x\right)\right)=f\,\left(H\,x+k\right) \end{array}$$

where *H* denotes the weight coefficient, *k* is the bias term, and the function *f* mostly uses the sigmoid, tanh, and ReLU functions. Core complex multilayer perceptron can contain several hidden layers. After the experiments, an MLP model with the following structure was selected: each hidden layer used a linear function, the input was the final embedding *Z*, and the output was the probability vector of MCI and NMCI. In the experiments, we adopted ReLU as the activation function and employed cross-entropy loss to supervise the learning of the multimodal brain network topological features.

### 2.5 Validation

To evaluate the performance of the different classification methods, we used a 5-fold cross-validation strategy to compute the classification accuracy (ACC), sensitivity (SEN), specificity (SPE), precision (PRE), recall (REC), F-measure (F1), and AUC. Specifically, five approximately equally sized, mutually exclusive subsets were partitioned from the entire dataset, four of which were used for training and the remaining for testing. Each algorithm was applied to the MCI recognition task, where the MCI dataset contained two classification labels (MCI group and NMCI group), which we considered a binary task to determine whether the subject had cognitive impairment.

### 2.6 Statistical analysis

Fisher’s exact test, independent samples *t*-test, and Mann–Whitney U test were used to determine whether there were statistically significant differences between the groups. Statistical analyses of demographic characteristics and neurobehavioural assessment results were performed using the Statistical Package for the Social Sciences, version 25 (SPSS 25, Chicago, Illinois, USA), with a significance level set at *p* < 0.05.

## 3 Experiment results

### 3.1 Multimodal classification

**FIGURE 3:**
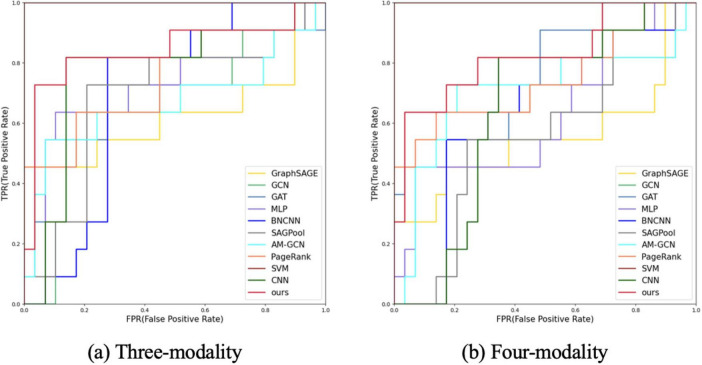
ROC curve analysis of different modes and different methods. **(A)** Three-modality analysis (fMRI, DTI, and 3D-T1WI data). **(B)** Four-modality analysis (rs-fMRI, DTI, 3D-T1WI, and demographic characteristics).

**FIGURE 4:**
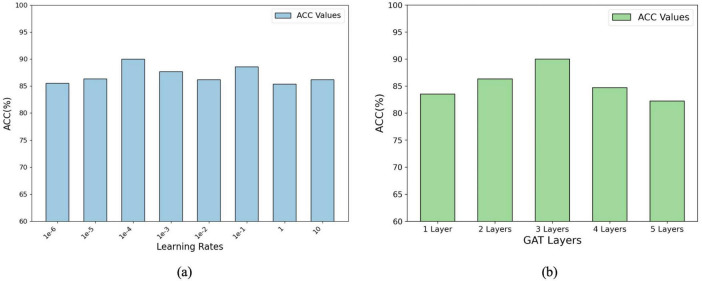
Parameter analysis of the model’s performance: **(A)** Accuracy (ACC%) with varying learning rates. **(B)** Accuracy (ACC%) with different numbers of GAT layers, showing optimal configurations for improved performance.

To validate the effectiveness of the proposed method, we compared it with the following ten FBN methods: GraphSAGE (Hamilton et al., 2017), GCN (Kipf and Welling, 2017), GAT (Veličković et al., 2018), MLP (Rosenblatt, 1958, Zhou et al., 2023), BrainNetCNN (Kawahara et al., 2017), SAGPool (Lee et al., 2019), AM-GCN (Wang et al., 2020), PageRank (Page et al., 1999), SVM (Cortes and Vapnik, 1995), and CNN (Lecun et al., 1998), Cross-GNN (Yang et al., 2024), and RH-BrainFS (Ye et al., 2023). The classification performance results of the different brain network construction methods are presented in Tables 2, 3. The evaluation metrics were ACC, PRE, REC, F1, AUC, SEN, and SPE. The best results are shown in bold. In addition, we plotted the ROC curves of the proposed methods and compared them (Figures 3, 4).

**TABLE 2:** Comparative experimental results based on rs-fMRI, DTI, and 3D-T1WI data.

Method	MCI vs. NMCI						
	**ACC**	**PRE**	**REC**	**F1**	**AUC**	**SEN**	**SPE**
GraphSAGE	80.00	53.33	46.67	46.00	70.50	46.67	96.67
GCN	70.00	59.24	83.33	61.33	57.57	83.33	65.24
GAT	82.50	53.33	53.33	53.33	75.00	53.33	96.00
MLP	80.00	63.33	53.33	54.00	69.67	53.33	92.67
BrainNetCNN	75.00	50.00	56.67	51.33	57.00	56.67	80.67
SAGPool	67.50	52.00	46.67	40.48	59.00	46.67	76.67
AM-GCN	77.50	53.33	46.67	48.10	61.00	46.67	84.67
PageRank	85.00	80.00	40.00	52.67	65.67	40.00	92.67
SVM	60.00	43.75	63.64	51.85	42.67	63.64	68.97
CNN	70.00	59.24	83.33	61.33	57.57	83.33	65.24
Cross-GNN	80.00	66.67	43.75	46.67	63.91	43.75	96.88
RH-BrainFS	80.00	66.67	39.58	42.50	69.49	39.58	96.88
Ours	87.50	91.67	83.33	82.50	87.50	83.33	96.88

ACC, accuracy; PRE, precision; REC, recall; F1, F-measure; AUC, area under the curve; SEN, sensitivity; SPE, specificity.

**TABLE 3:** Comparative experimental results based on rs-fMRI, DTI, 3D-T1WI, and demographic characteristic data.

Method	MCI vs. NMCI							
	**ACC**	**PRE**	**REC**	**F1**	**AUC**	**SEN**	**SPE**	***p*-value**
GraphSAGE	85.00	80.00	53.33	62.67	70.00	53.33	96.00	0.542
GCN	75.00**	68.00	63.33	56.10	67.14	63.33	83.14	0.048
GAT	82.50*	48.33	60.00	53.14	70.00	60.00	92.67	0.133
MLP	82.50*	66.67	56.67	60.00	77.00	56.67	92.00	0.140
BrainNetCNN	72.50**	41.90	80.00	54.67	66.14	80.00	65.81	0.033
SAGPool	77.50**	50.00	70.00	56.67	70.33	70.00	78.00	0.043
AM-GCN	82.50	70.00	50.00	52.67	59.33	50.00	93.33	0.176
PageRank	85.00	80.00	45.00	54.67	73.15	45.00	87.14	0.542
SVM	75.00**	62.50	45.45	52.63	78.00	45.45	89.66	0.047
CNN	85.00	60.00	80.00	67.62	73.33	80.00	85.33	0.360
Cross-GNN	85.00	66.67	52.08	53.93	84.89	52.08	96.87	0.255
RH-BrainFS	81.25*	16.67	16.67	16.67	24.05	16.67	95.00	0.149
Ours	90.00	91.67	81.67	83.75	91.49	81.67	96.88	–

ACC, accuracy; PRE, precision; REC, recall; F1, F-measure; AUC, area under the curve; SEN, sensitivity; SPE, specificity. *p*-value between the comparison methods and the proposed method:

*indicating *p* ≤ 0.15,

**indicating *p* ≤ 0.05.

First, we tested the performance of our multimodal classification method using rs-fMRI, DTI, and 3D-T1WI for differentiating MCI from NMCI. Table 2 shows the classification with our multimodal method compared with other methods. In the MCI and NMCI classification tasks, the ACC of the three-modal approach was 87.50% (SEN: 83.33%; SPE: 96.88%). The AUC value was 0.8750, which was higher than the AUC values of the other ten methods, indicating that the three-modal approach is effective and has good generalis ability in MCI diagnosis.

The performances of the different methods in the MCI and NMCI classification tasks after adding demographic characteristics are shown in Table 3. When demographic characteristics were combined with the three-modal approach, the ACC of the four-modal classification reached 90.00% (sensitivity = 81.67% and specificity = 96.88%) and the AUC reached 0.9149, which were higher than those of the other 10 methods. Compared with using only the three-modal approach, except for a slight decrease in SEN and REC, the ACC, F1, and AUC values improved, with a 3.99% increase in the AUC value (Table 1). The *p*-values presented in Table 3 highlight the statistical significance of the proposed method compared with the existing approaches. Specifically, the *p*-values for comparisons with the GCN (*p* = 0.048), BrainNetCNN (*p* = 0.033), SAGPool (*p* = 0.043), and SVM (*p* = 0.047) were all less than 0.05, indicating that the improvements achieved by the proposed method were statistically significant. Furthermore, when compared with the RH-BrainFS (*p* = 0.149), GAT (*p* = 0.133), and MLP (*p* = 0.140), the *p*-values are < 0.15, demonstrating a trend toward significance. These results substantiate the superior performance of the proposed method, while underscoring the reliability of the observed improvements across various metrics. The experimental results indicated that the four-modal approach had superior classification ability for MCI. In addition, from the experimental results, we can found that rs-fMRI, DTI, 3D-T1WI, and demographic features complemented each other and jointly improved ACC of MCI diagnosis. Extensive experimental results further demonstrate that our method is effective and outperforms other algorithms.

Figure 3 shows the ROC curves of the proposed method and the comparison methods, where the proposed method is represented by a thick red curve. These graphs show that the ROC curves of the comparison methods are mostly located below and to the right of the ROC curve of our method, whereas the area under the ROC curve of the comparative methods is significantly smaller than that of our method.

In general, from these tables, we find that our method achieved the optimal performance in all metrics compared with the comparison methods. This is because the proposed method effectively captured the complex topological information of brain networks and fused complementary information from the different modalities.

Figure 4 illustrates the impact of the learning rate and number of GAT layers on the model performance (measured by percentage accuracy, ACC%). In panel (a), the accuracy initially improves as the learning rate increases, before stabilizing or declining, indicating that selecting an appropriate learning rate is crucial for effective model optimisation. The optimal learning rate was determined using grid search over the range {1e-6, 1e-5, 1e-4, 1e-3, 1e-2, 1e-1, 1, 10}. Panel (b) shows that the accuracy increases with the number of GAT layers up to a certain point, after which it starts to decline. The optimal number of GAT layers was determined through a grid search within the range of {1, 2, 3, 4, 5}. These results suggest that while additional layers can enhance the representational capacity of the model, an excessive number of layers may lead to overfitting or gradient-related issues. These findings emphasize the importance of systematically tuning hyperparameters such as the learning rate and layer number to balance model performance and complexity and ensure optimal outcomes during training.

In addition, we evaluated the computational efficiency of the proposed approach to highlight its practicality. The experimental results indicated that the model incorporated 9.1579 million trainable parameters and required 9.8855 million floating-point operations per forward pass. These values reflect the lightweight nature of the framework, allowing it to deliver high diagnostic accuracy with minimal computational demands. This balance between performance and efficiency underscores its potential for real-world applications, particularly in resource-constrained environments.

### 3.2 Ablation experiments

Different modalities provide complementary information for the diagnosis of cognitive impairment and other diseases, allowing the distinguishing features of the disease to be captured from multiple perspectives. We further studied the effectiveness of using discriminative information provided by multimodal data to enhance the diagnostic performance for diseases involving cognitive impairment. First, the existing architecture of the model was maintained throughout the analysis to ensure consistency. We then removed the rs-fMRI, DTI, 3D-T1WI, and demographic characteristic data to diagnose diseases with cognitive impairment while retaining the rs-fMRI, DTI, 3D-T1WI, and demographic characteristics for diagnosis. Finally, we integrated the four modal datasets to validate the improvement in multimodal information and the role of each modality. The results of the ablation experiments are listed in Table 4, which shows an improvement in the classification performance compared to the fusion of all four modalities.

**TABLE 4:** Results of the ablation experiments.

Modality	MCI vs. NMCI						
	**ACC**	**PRE**	**REC**	**F1**	**AUC**	**SEN**	**SPE**
w/o rs-fMRI	82.50	53.33	45.00	44.00	65.95	45.00	96.67
w/o DTI	87.50	85.42	77.08	80.42	88.39	77.08	92.26
w/o 3D-T1WI	87.50	73.33	70.00	69.33	81.67	70.00	90.00
w/o demographic characteristics	87.50	75.00	63.33	66.48	75.67	63.33	96.00
rs-fMRI	80.00	46.67	70.00	54.29	76.14	70.00	82.95
DTI	85.00	66.67	50.00	52.50	53.13	50.00	91.67
3D-T1WI	87.50	75.00	47.92	55.00	77.43	47.92	96.67
Demographic characteristics	85.00	92.00	66.67	71.67	68.67	66.67	92.00
Ours	90.00	91.67	81.67	83.75	91.49	81.67	96.88

w/o, without.

### 3.3 t-SNE visualization

**FIGURE 5:**
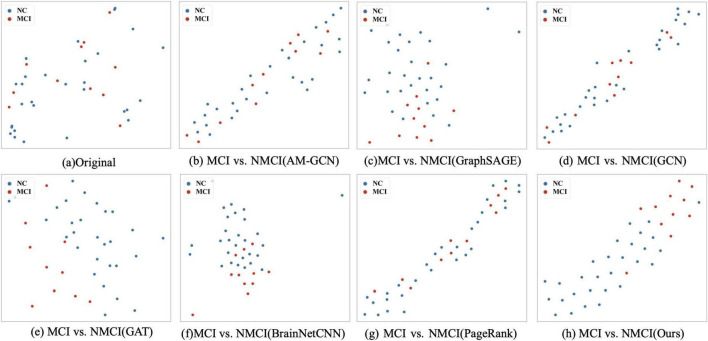
t-SNE visualizations of the different methods: **(A)** The scattered distribution of the original sample in the representation space. **(B–G)** The representation space of different methods on tasks MCI vs. NMCI, and **(H)** is the distribution of the features of our proposed methods in the representation space. In particular, the t-SNE scatter plot in our method exhibits a clearer separation between classes, demonstrating superior clustering and boundary delineation compared to the other methods.

To validate the feature extraction capability of the proposed method, we compared the learned features extracted using different methods. First, we reduced the dimensionality of the representation vectors using the t-SNE method (Maaten and Hinton, 2008), which is commonly used to visualize high-dimensional data by mapping it into a lower-dimensional space, typically two or three dimensions, while preserving the local structure of the data. We projected the two-dimensional vectors onto a public space for visualization, as shown in Figure 5. Further, we visualized the results of the original sample data and comparison methods, as shown in Figures 5A–G, respectively. According to the experimental results, our method demonstrates a higher clustering of samples from the same class and clear boundaries between different categories of samples.

### 3.4 Discriminative ROIs

In addition to the diagnostic performance of the classification, significant changes in brain regions and connections can be used to evaluate the performance of the brain network. Because not all ROIs are closely related to cognitive impairment, we used our proposed method to identify the most discriminative ROIs to understand brain abnormalities. By calculating the significant alterations in connectivity (SAC), we demonstrated local differences in brain networks. SAC quantifies the changes in connectivity strength between specific brain regions by comparing the brain network structures of different groups. Specifically, SAC is calculated by measuring the difference in connectivity values between corresponding brain regions across groups. Specifically, we applied a non-negative elastic net to measure the important brain regions in the brain network embedded prior to classification for each subject. We subsequent visualized the 20 most relevant ROIs and the top ten connections between them in the NMCI and MCI task.

**FIGURE 6:**
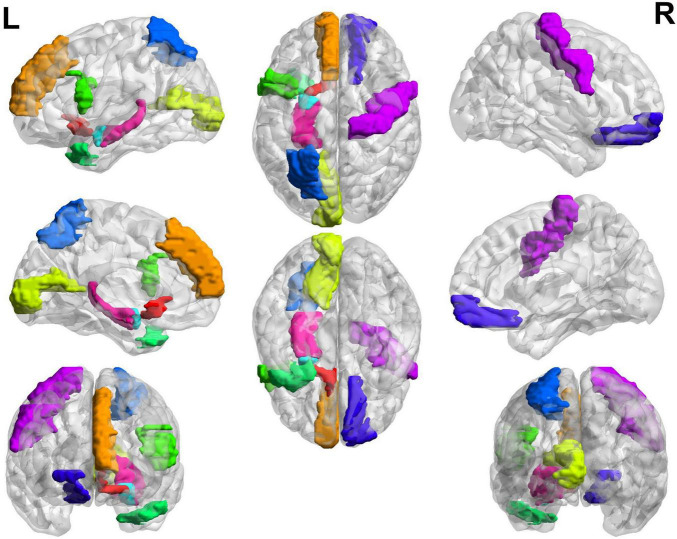
Top 20 SACs between the MCI and NMCI groups. Brain regions with significant structural changes include Olfactory_L, Frontal_Sup_Medial_L, Parietal_Sup_L, Temporal_Pole_Mid_L, Frontal_Inf_Tri_R, Amygdala_L, Hippocampus_L, Frontal_Sup_Orb_R, and Paracentral_Lobule_L. Different brain regions are represented by different colors.

The top 20 brain regions with significant weights in the MCI and NMCI classifications are presented in Table 5 and Figure 6. The English abbreviations corresponding to the Chinese and English names of the brain regions are listed in the Supplementary Appendix 1. The larger the weight of a brain region, the more likely it was to undergo significant changes. Brain regions with significant structural changes found using this method were confirmed to be associated with MCI, including Olfactory_L, Frontal_Sup_Medial_L, Parietal_Sup_L, Temporal_Pole_Mid_L, Frontal_Inf_Tri_R, Amygdala_L, Hippocampus_L, Frontal_Sup_Orb_R, and Paracentral_Lobule_L.

**TABLE 5:** Top 20 SACs between the MCI and NMCI groups.

No	Brain region	Weight
1	Olfactory_L	2.0813
2	Frontal_Sup_Medial_L	0.8685
3	Calcarine_L	0.8178
4	Frontal_Inf_Oper_L	0.8059
5	Temporal_Pole_Mid_L	0.7753
6	Amygdala_L	0.7066
7	Parietal_Sup_L	0.6641
8	Frontal_Sup_Orb_R	0.5934
9	Precentral_R	0.5196
10	Hippocampus_L	0.5095
11	Frontal_Sup_Orb_L	0.4984
12	Occipital_Sup_L	0.4941
13	Frontal_Inf_Tri_R	0.4638
14	Paracentral_Lobule_L	0.4350
15	Frontal_Mid_R	0.4264
16	Caudate_R	0.3770
17	Parietal_Sup_R	0.3687
18	Temporal_Pole_Sup_R	0.3671
19	Cuneus_L	0.3148
20	Frontal_Mid_L	0.2635

**FIGURE 7:**
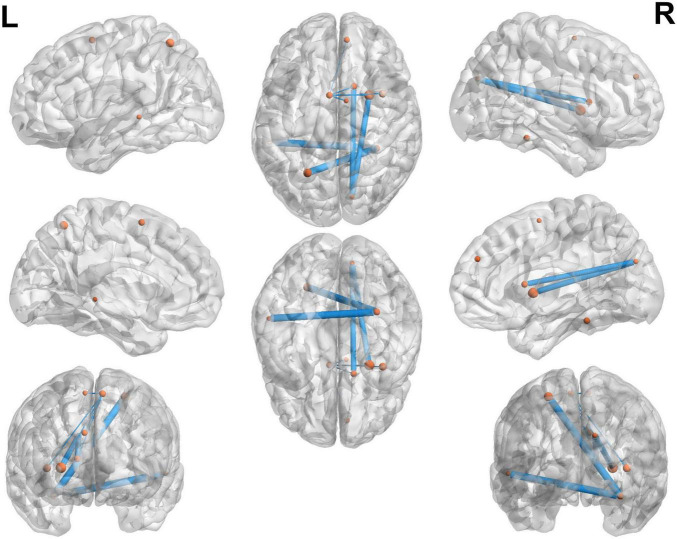
Top 10 brain connections between the MCI and NMCI groups. The 10 brain connections with higher weights in the MCI and NMCI classifications are represented by yellow dots. These connections include Fusiform_R—Precentral_R, Fusiform_R—Occipital_Sup_L, Fusiform_R—Temporal_Mid_L, Cuneus_R—Supp_Motor_Area_R, Cuneus_R—Frontal_Sup_Orb_L, Fusiform_R—Frontal_Inf_Orb_L, Cuneus_R—Putamen_R, Cuneus_R—Insula_R, Supp_Motor_Area_L—Putamen_R, and Supp_Motor_Area_L—Frontal_Sup_Orb_L.

In the classification of MCI and NMCI, the 10 brain connections with higher weights, as shown in Table 6 and Figure 7, the abnormal connections were distributed throughout the brain, showing asymmetry between the left and right hemispheres. Specifically, the connections between the Fusiform_R, Cuneus_R, and Supp_Motor_Area_L, and other brain regions showed significant changes.

**TABLE 6:** Top 10 brain connections between the MCI and NMCI groups.

NO	Connection	Weight
1	Fusiform_R—Precentral_R	91.2125
2	Fusiform_R—Occipital_Sup_L	89.9365
3	Fusiform_R—Temporal_Mid_L	85.2252
4	Cuneus_R—Supp_Motor_Area_R	82.1950
5	Cuneus_R—Frontal_Sup_Orb_L	79.3418
6	Fusiform_R—Frontal_Inf_Orb_L	75.7217
7	Cuneus_R—Putamen_R	74.0230
8	Cuneus_R—Insula_R	61.9906
9	Supp_Motor_Area_L—Putamen_R	39.3506
10	Supp_Motor_Area_L—Frontal_Sup_Orb_L	39.1733

## 4 Discussion

MCI increasingly poses a significant economic and social burden on patients with unilateral MCA steno-occlusive disease. Early diagnosis of MCI is of paramount importance for maximizing treatment effectiveness. Therefore, in this study, we proposed a multimodal imaging technique based on rs-fMRI, DTI, 3D-T1WI, and demographic characteristics for the identification of MCI in patients with unilateral MCA steno-occlusive disease. This technique obtains the most discriminative features of MCI and NMCI by combining the information of multiple modalities, obtains a better classification result, and improves the ACC of diagnosis. Efficient automated diagnosis facilitates early and accurate detection of MCI and timely intervention and treatment to delay or prevent disease progression.

Overall, the results of the present study indicate that the discrepant regions are widely distributed throughout the brain, including the Olfactory_L, Frontal_Sup_Medial_L, Calcarine_L, Frontal_Inf_Oper_L, Temporal_Pole_Mid_L, and Amygdala_L (Whitwell et al., 2007; Bozzali et al., 2006; Hämäläinen et al., 2007; Chen et al., 2020). Previous studies have shown that intracranial stenosis (ICS) is associated with cognitive impairment, independent of vascular risk factors. This association may be attributed to subtle cortical and subcortical ischaemic damage, including increased resistance and reduced vascular reactivity of small vessels, or to reduction in anatomic connectivity and perfusion deficits secondary to ICS (Hilal et al., 2015; Hilal et al., 2017). All of above regions are commonly involved in MCI-related pathology; as such, their involvement may represent the early features of MCI pathology. The frontal lobe has the largest number of differential brain regions, which primarily include the centers of higher cortical nerves and motor speech centers. Several studies have shown that reduced frontal lobe volume compared to the normal group can be found at the MCI stage (Yener et al., 2016). In addition, the present study found that the inner olfactory cortex had the greatest weight and showed the most significant differences. Consistent with our findings, the olfactory decline caused by lesions in this region was previously found to be highly discriminatory for MCI (Roberts et al., 2016). Previous studies have shown that volume loss in the internal olfactory cortex is greater than hippocampal volume loss in patients with MCI (Pennanen et al., 2004), suggesting that the volume of the internal olfactory cortex is better able to differentiate between patients with MCI and controls than the hippocampal volume. One meta-analysis found a reduced gray matter volumes in patients with MCI compared to controls, most notably in the hippocampus, parahippocampal gyrus, and amygdala (Raine and Rao, 2022). In addition, at the neuropsychological level, an 18F-AV-1451 PET imaging study showed that tau proteins accumulated only in the internal olfactory cortex of patients with MCI (Cho et al., 2016).

This study also found that the brain connections that distinguished MCI from NMCI were primarily located in the fusiform R, cuneus R, and supplementary motor area L. The fusiform gyrus, the largest component of the ventral temporal cortex, plays a key role in visual categorization and is associated with high-level tasks related to visual processing (Grill-Spector and Weiner, 2014; Palejwala et al., 2020). In one analysis based on fMRI data, Spagna et al. (2021) found that the fusiform gyrus is associated with visual images, and that damage to it causes deficits in the construction of visual images, which in turn leads to a decrease in visual memory capacity (Bartolomeo et al., 2020; Tabi et al., 2022). The cuneus is a part of the occipital lobe, forming the primary visual cortex along with the surrounding cortex, which is involved in the integration of visual space and visual motion, and plays an important role in non-visual functions such as language and memory (Tanglay et al., 2022; Palejwala et al., 2021). The supplementary motor area forms part of the secondary motor system and is mainly responsible for somatosensory motor functions, with extensive fiber connections to the cingulate gyrus and frontal lobe, playing a key role in the integration of functions as well as emotions, behaviors, and cognitive functions (Leisman et al., 2016). Ye et al. (2019) used multivariate distance matrix regression to investigate abnormal connectivity patterns of the SBN, identifying abnormalities in the supplementary motor area in patients with MCI. These findings are consistent with those of previous studies, showing that brain regions and connections may play important roles in the development of cognitive impairment in patients with unilateral MCA occlusion. Exploring the exact mechanism of these regions in cognitive dysfunction will help to enrich our knowledge of the developmental process of this disease, and to provide a scientific basis for future clinical practice.

This study adopted a fully automatic brain segmentation software, which has high automation, high data consistency, fast analysis, and high accuracy and can ensure data analysis of large sample sizes, thereby ensuring the reliability of the research results. Further, we propose a framework integrating the topological features of a multimodal (four modalities) FBN and SBN, and design a multi-channel graph attention network to extract the topological features of multimodal brain networks to allow using attention mechanisms. This proposed model can detect subtle pathological physiological abnormalities in the brain more accurately than a single modality, thus improving diagnostic efficiency. Intracranial arterial stenosis has attracted significant attention owing to its high incidence in the Asian population and the potential for long-term adverse events such as stroke. Many studies have further focused on the effects of carotid artery stenosis on cognition. However, studies on the impact of intracranial vessels on cognition are rare, and no unified conclusions have yet been reached. As such, this study is innovative.

This study has some limitations. First, the small sample size and imbalance between the MCI and NMCI groups are limitations of the current study, limitations commonly attributed to single-center studies and disease specificity. We used statistical methods such as 5-fold cross-validation to minimize the impact and ensure the accuracy of the results. Second, we recognize the limitations of single-center studies and plan to increase the sample size in the future through collaborative multi-center studies to ensure that device parameters are consistent across centers to further improve the reliability and generalisability of the data. Third, we plan to conduct a long-term follow-up study to validate and extend the results of the current study. With a long-term follow-up, we can gain a deeper understanding of disease progression, observe the performance of the model at different time points, comprehensively assess its validity and generalisability, and predict future cognitive decline.

## 5 Conclusion

In this study, we propose a multimodality (four-modalities) FBN and SBN fusion framework that can effectively fuse rs-fMRI, DTI, 3D-T1WI, and demographic characteristic data. This framework can also embed the characteristics of different modalities into the fusion model, which can effectively extract complex and complementary topological structural information from the brain network. The experimental results show that our method not only achieves good performance in the diagnosis of MCI, but can also effectively identify disease-related brain areas and connections, which provides a promising prospect for the diagnosis of auxiliary brain diseases. Temporal changes in brain activity are extremely important in the analysis brain networks. Therefore, the investigation of brain networks’ spatiotemporal evolution, leveraging their dynamic transformational properties, stands as a promising research pathway for deepening our understanding of brain disease mechanisms. However, the proposed method primarily studies static brain networks and ignores the dynamic attributes of the brain. In future research, we will design methods to extract the spatio-temporal features of dynamic brain networks to comprehensively capture the evolutionary information of brain networks.

## Data Availability

The raw data supporting the conclusions of this article will be made available by the authors, without undue reservation.
