# Supplementary material for: Multimodal fusion model for diagnosing mild cognitive impairment in unilateral middle cerebral artery steno-occlusive disease
Source: Front Aging Neurosci. 2025 Feb 12;17:1527323. doi: 10.3389/fnagi.2025.1527323 (PMC11861546; doi:10.3389/fnagi.2025.1527323)
Supplement: Supplementary file 1 [file Data_Sheet_1.docx]

Appendix 1 The English abbreviations corresponding to the Chinese and English names of the brain regions

| Labels | Regions | Regions |
| --- | --- | --- |
| 1 | Precentral_L | Precental gyrus |
| 2 | Precentral_R | Precental gyrus |
| 3 | Frontal_Sup_L | Superior frontal gyrus, dorsolateral |
| 4 | Frontal_Sup_R | Superior frontal gyrus, dorsolateral |
| 5 | Frontal_Sup_Orb_L | Superior frontal gyrus, orbital part |
| 6 | Frontal_Sup_Orb_R | Superior frontal gyrus, orbital part |
| 7 | Frontal_Mid_L | Middle frontal gyrus |
| 8 | Frontal_Mid_R | Middle frontal gyrus |
| 9 | Frontal_Mid_Orb_L | Middle frontal gyrus, orbital part |
| 10 | Frontal_Mid_Orb_R | Middle frontal gyrus, orbital part |
| 11 | Frontal_Inf_Oper_L | Inferior frontal gyrus, opercular part |
| 12 | Frontal_Inf_Oper_R | Inferior frontal gyrus, opercular part |
| 13 | Frontal_Inf_Tri_L | Inferior frontal gyrus, triangular part |
| 14 | Frontal_Inf_Tri_R | Inferior frontal gyrus, triangular part |
| 15 | Frontal_Inf_Orb_L | Inferior frontal gyrus, orbital part |
| 16 | Frontal_Inf_Orb_R | Inferior frontal gyrus, orbital part |
| 17 | Rolandic_Oper_L | Rolandic operculum |
| 18 | Rolandic_Oper_R | Rolandic operculum |
| 19 | Supp_Motor_Area_L | Supplementary motor area |
| 20 | Supp_Motor_Area_R | Supplementary motor area |
| 21 | Olfactory_L | Olfactory cortex |
| 22 | Olfactory_R | Olfactory cortex |
| 23 | Frontal_Sup_Medial_L | Superior frontal gyrus, medial |
| 24 | Frontal_Sup_Medial_R | Superior frontal gyrus, medial |
| 25 | Frontal_Mid_Orb_L | Superior frontal gyrus, medial orbital |
| 26 | Frontal_Mid_Orb_R | Superior frontal gyrus, medial orbital |
| 27 | Rectus_L | Gyrus rectus |
| 28 | Rectus_R | Gyrus rectus |
| 29 | Insula_L | Insula |
| 30 | Insula_R | Insula |
| 31 | Cingulum_Ant_L | Anterior cingulate and paracingulate gyri |
| 32 | Cingulum_Ant_R | Anterior cingulate and paracingulate gyri |
| 33 | Cingulum_Mid_L | Median cingulate and paracingulate gyri |
| 34 | Cingulum_Mid_R | Median cingulate and paracingulate gyri |
| 35 | Cingulum_Post_L | Posterior cingulate gyrus |
| 36 | Cingulum_Post_R | Posterior cingulate gyrus |
| 37 | Hippocampus_L | Hippocampus |
| 38 | Hippocampus_R | Hippocampus |
| 39 | ParaHippocampal_L | Parahippocampal gyrus |
| 40 | ParaHippocampal_R | Parahippocampal gyrus |
| 41 | Amygdala_L | Amygdala |
| 42 | Amygdala_R | Amygdala |
| 43 | Calcarine_L | Calcarine fissure and surrounding cortex |
| 44 | Calcarine_R | Calcarine fissure and surrounding cortex |
| 45 | Cuneus_L | Cuneus |
| 46 | Cuneus_R | Cuneus |
| 47 | Lingual_L | Lingual gyrus |
| 48 | Lingual_R | Lingual gyrus |
| 49 | Occipital_Sup_L | Superior occipital gyrus |
| 50 | Occipital_Sup_R | Superior occipital gyrus |
| 51 | Occipital_Mid_L | Middle occipital gyrus |
| 52 | Occipital_Mid_R | Middle occipital gyrus |
| 53 | Occipital_Inf_L | Inferior occipital gyrus |
| 54 | Occipital_Inf_R | Inferior occipital gyrus |
| 55 | Fusiform_L | Fusiform gyrus |
| 56 | Fusiform_R | Fusiform gyrus |
| 57 | Postcentral_L | Postcentral gyrus |
| 58 | Postcentral_R | Postcentral gyrus |
| 59 | Parietal_Sup_L | Superior parietal gyrus |
| 60 | Parietal_Sup_R | Superior parietal gyrus |
| 61 | Parietal_Inf_L | Inferior parietal, but supramarginal and angular gyri |
| 62 | Parietal_Inf_R | Inferior parietal, but supramarginal and angular gyri |
| 63 | SupraMarginal_L | Supramarginal gyrus |
| 64 | SupraMarginal_R | Supramarginal gyrus |
| 65 | Angular_L | Angular gyrus |
| 66 | Angular_R | Angular gyrus |
| 67 | Precuneus_L | Precuneus |
| 68 | Precuneus_R | Precuneus |
| 69 | Paracentral_Lobule_L | Paracentral lobule |
| 70 | Paracentral_Lobule_R | Paracentral lobule |
| 71 | Caudate_L | Caudate nucleus |
| 72 | Caudate_R | Caudate nucleus |
| 73 | Putamen_L | Lenticular nucleus, putamen |
| 74 | Putamen_R | Lenticular nucleus, putamen |
| 75 | Pallidum_L | Lenticular nucleus, pallidum |
| 76 | Pallidum_R | Lenticular nucleus, pallidum |
| 77 | Thalamus_L | Thalamus |
| 78 | Thalamus_R | Thalamus |
| 79 | Heschl_L | Heschl gyrus |
| 80 | Heschl_R | Heschl gyrus |
| 81 | Temporal_Sup_L | Superior temporal gyrus |
| 82 | Temporal_Sup_R | Superior temporal gyrus |
| 83 | Temporal_Pole_Sup_L | Temporal pole: superior temporal gyrus |
| 84 | Temporal_Pole_Sup_R | Temporal pole: superior temporal gyrus |
| 85 | Temporal_Mid_L | Middle temporal gyrus |
| 86 | Temporal_Mid_R | Middle temporal gyrus |
| 87 | Temporal_Pole_Mid_L | Temporal pole: middle temporal gyrus |
| 88 | Temporal_Pole_Mid_R | Temporal pole: middle temporal gyrus |
| 89 | Temporal_Inf_L | Inferior temporal gyrus |
| 90 | Temporal_Inf_R | Inferior temporal gyrus |
